# Two new species in the *Echinoderes coulli* group (Echinoderidae, Cyclorhagida, Kinorhyncha) from the Ryukyu Islands, Japan

**DOI:** 10.3897/zookeys.382.6761

**Published:** 2014-02-20

**Authors:** Hiroshi Yamasaki, Shinta Fujimoto

**Affiliations:** 1 Department of Chemistry, Biology & Marine Science, Faculty of Science, University of the Ryukyus, Senbaru 1, Nishihara, Nakagami, Okinawa 903-0213, Japan; 2 Department of Zoology, Division of Biological Science, Graduate School of Science, Kyoto University, Kitashirakawa-Oiwakecho, Sakyo-ku, Kyoto 606-8502, Japan

**Keywords:** *Echinoderes*, Kinorhyncha, Meiofauna, Taxonomy, Okinawa, Ishigaki

## Abstract

Two new species belonging to the *Echinoderes coulli* group are described with their external morphologies and sequences of nuclear 18S rRNA and 28S rRNA genes, and mitochondrial COI gene. The first species, *Echinoderes komatsui* **sp. n.**, is characterized by absence of acicular spines, and presence of lateroventral tubules on segments 5 and 8, laterodorsal tubules on segment 10, inverted triangle or wide oval shaped large sieve plates, lateral terminal accessory spines in female, and short tips of ventral pectinate fringe on segment 10. The second species, *Echinoderes hwiizaa* **sp. n.**, is characterized by absence of acicular spines, and presence of lateroventral tubules on segments 5 and 7–9, midlateral tubules on segment 8, laterodorsal tubules on segment 10, large narrow oval shaped sieve plates on segment 9, and thick, short and blunt lateral terminal spines about 10–15% of trunk length. The diagnostic characters and key to species of *E. coulli* group are provided as well.

## Introduction

*Echinoderes* is the most species-rich genus in the marine phylum Kinorhyncha. At present, 82 *Echinoderes* species have been reported worldwide, from the intertidal zone to abyssal depths and from polar to tropical regions (Sørensen and Pardos 2008, Herranz et al. 2013, Neuhaus 2013, Sørensen 2013, 2014). Only a few species have been reported from brackish waters and most of these belong to the *Echinoderes coulli* species group.

The *Echinoderes coulli* group was proposed for the first time by Ostmann et al. (2012), as comprising species adapted to highly fluctuating estuarine habitats. Currently the group contains seven species (*Echinoderes applicitus* Ostmann et al., 2012; *Echinoderes coulli* Higgins, 1977; *Echinoderes marthae* Sørensen, 2014; *Echinoderes maxwelli* Omer-Cooper, 1957; *Echinoderes ohtsukai* Yamasaki & Kajihara, 2012; *Echinoderes rex* Lundbye et al., 2011; and *Echinoderes teretis* Brown, 1999) (Omer-Cooper 1957, Higgins 1977, Brown 1985, Adrianov and Malakhov 1999, Lundbye et al. 2011, Ostmann et al. 2012, Yamasaki and Kajihara 2012, Sørensen 2014). These species share the following morphological features: (1) middorsal spines are absent, or reduced to a short spine and occur only on segment 4; (2) lateroventral acicular spines are absent, or if present, very short and occur on segments 6 and 7; (3) lateral tubules are present at least on segments 5 and 8; (4) relatively large sieve plates consisting of a sieve area and a posterior pore are present; (5) lateral terminal accessory spines are absent in both sexes. The large sieve plates, which function in osmotic regulation, appear to be adaptive to estuarine habitats.

Among the seven species in the *Echinoderes coulli* group, only *Echinoderes rex* has not been reported from an estuarine environment, but instead inhabits subtidal marine waters (Lundbye et al. 2011). Two alternative hypotheses might explain the origin of adaptations to brackish water environments: (1) the adaptive characters arose only once, and *Echinoderes rex* has secondarily returned to a fully marine environment, or (2) adaptive characters arose several times independently in the *Echinoderes coulli* group (Ostmann et al. 2012, Yamasaki and Kajihara 2012). No phylogenetic analysis has been performed to determine which hypothesis is correct yet.

In this paper we describe two new species of the *Echinoderes coulli* group collected from the Ryukyu Islands, southern Japan. In addition to the morphological descriptions, we include the sequences of three genes for each of these species. We also summarize the morphological diagnostic characters for the group and provide a key to species.

## Materials and methods

**Figure 1.**
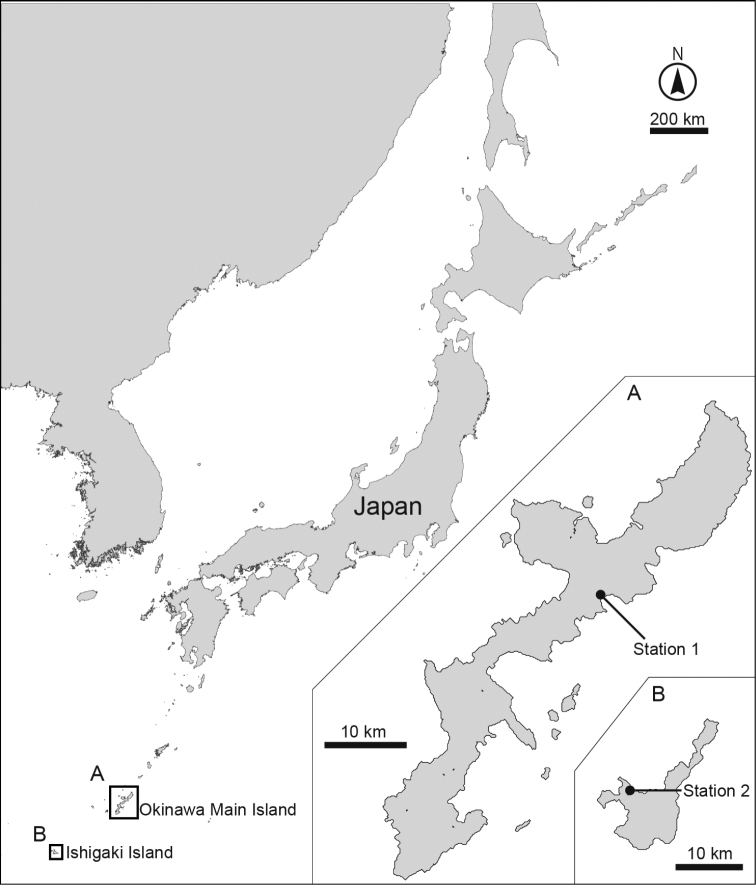
Maps showing the sampling sites. **A** Close-up of the Okinawa main island and **B** Ishigaki island.

Sediment samples were taken at two stations by hand at low tide (Fig. 1). Station 1 is an intertidal flat with a mangrove area in Oura Bay, Okinawa Island, Japan (26°33.35'N, 128°2.57'E); samples were collected on 26 May 2013 and 8 July 2013. Station 2 is an intertidal flat in Kabira Bay, Ishigaki Island, Japan (24°27.58'N, 124°8.57'E); samples were collected on 23 June 2013. All samples consisted of mud mixed with sand, without black sulfide sediments.

Kinorhynchs were extracted from the samples by using the bubbling and blot method (Higgins 1988, Sørensen and Pardos 2008). Extracted animals including kinorhynchs were washed with fresh water and preserved in 99% EtOH. In the laboratory, kinorhynch specimens were sorted under a stereomicroscope. Some specimens were used for DNA extraction. The others were observed by light microscopy (LM) or scanning electron microscopy (SEM).

Total genomic DNA was extracted from selected single individuals with a DNeasy Tissue Kit (Qiagen, Tokyo), following the protocol of Yamasaki et al. (2013). After DNA extraction, the exoskeleton of each specimen was mounted for LM as described below. Parts of the nuclear 18S and 28S rRNA genes and the mitochondrial COI gene were amplified by PCR using primer sets 18S-F1/18S-R9 for 18S; 28S-01/28Sr, 28Sf/28S-3KR, and 28S-2KF/28jj-3’ for 28S; and LCO1490/HCO2198 for COI (see Table 1 for primer references and sequences). PCR conditions for 18S and 28S were 95 °C for 1 min; 35 cycles of 95 °C for 30 sec, 45 °C for 1 min 30 sec, and 72 °C for 3 min; and 72 °C for 7 min. Conditions for COI were 95 °C for 1 min; 35 cycles of 95 °C for 30 sec, 45 °C for 1 min 30 sec, and 72 °C for 90 sec; and 72 °C for 7 min. All nucleotide sequences were determined by direct sequencing with a BigDye Terminator Kit ver. 3.1 (Life Technologies, Co., USA) and a 3730 DNA Analyzer (Life Technologies, Co., USA). Sequence fragments were assembled by using MEGA 5 (Tamura et al. 2011). After assembly, sequences were deposited in GenBank under accession numbers AB899164–AB899171.

**Table 1.** List of PCR and cycle sequencing (CS) primers used in this study.

Gene	Primer name	Reaction	Primer sequence (in 5’–3’ direction)	Direction	Source
18S rRNA	F1	PCR & CS	TACCTGGTTGATCCTGCCAG	Forward	Yamaguchi and Endo (2003)
	R9	PCR & CS	GATCCTTCCGCAGGTTCACCTAC	Reverse	Yamaguchi and Endo (2003)
	F2	CS	CCTGAGAAACGGCTRCCACAT	Forward	Yamaguchi and Endo (2003)
	F3	CS	GYGRTCAGATACCRCCSTAGTT	Forward	Yamaguchi and Endo (2003)
	F4	CS	GGTCTGTGATGCCCTYAGATGT	Forward	Yamaguchi and Endo (2003)
	R6	CS	TYTCTCRKGCTBCCTCTCC	Reverse	Yamaguchi and Endo (2003)
	R7	CS	GYYARAACTAGGGCGGTATCTG	Reverse	Yamaguchi and Endo (2003)
	R8	CS	ACATCTRAGGGCATCACAGACC	Reverse	Yamaguchi and Endo (2003)
28S rRNA	28S-01	PCR & CS	GACTACCCCCTGAATTTAAGCAT	Forward	Kim et al. (2000)
	28Sr	PCR & CS	ACACACTCCTTAGCGGA	Reverse	Luan et al. (2005)
	28Sf	PCR & CS	TGGGACCCGAAAGATGGTG	Forward	Luan et al. (2005)
	28S-3KR	PCR & CS	CCAATCCTTTTCCCGAAGTT	Reverse	Yamasaki et al. (2013)
	28S-2KF	PCR & CS	TTGGAATCCGCTAAGGAGTG	Forward	Yamasaki et al. (2013)
	28jj-3’	PCR & CS	AGTAGGGTAAAACTAACCT	Reverse	Palumbi (1996)
	28S-n05R	CS	CTCACGGTACTTGTTCGCTAT	Reverse	Yamasaki et al. (2013)
	28SR-01	CS	GACTCCTTGGTCCGTGTTTCAAG	Reverse	Kim et al. (2000)
	28S-15R	CS	CGATTAGTCTTTCGCCCCTA	Reverse	Yamasaki et al. (2013)
	28S-3KF	CS	AGGTGAACAGCCTCTAGTCG	Forward	Yamasaki et al. (2013)
	28v-5’	CS	AAGGTAGCCAAATGCCTCATC	Forward	Palumbi (1996)
	28S-42F	CS	GAGTTTGACTGGGGCGGTA	Forward	Yamasaki et al. (2013)
COI	LCO1490	PCR & CS	GGTCAACAAATCATAAAGATATTGG	Forward	Folmer et al. (1994)
	HCO2198	PCR & CS	TAAACTTCAGGGTGACCAAAAAATCA	Reverse	Folmer et al. (1994)

Specimens for LM were transferred into dehydrated glycerin to replace the ethanol with glycerin and were then mounted in Fluoromount G® between two cover slips attached to a plastic H-S slide. They were observed, sketched, and photographed with an Olympus BX51 microscope equipped with a Nikon DS-Fi1c camera and a drawing tube. Line illustrations were drawn in Adobe Illustrator CS5, based on scanned camera lucida drawings of mounted specimens. Measurements were made with a Nikon DS-L3 camera control unit.

Specimens for SEM were immersed in 100% butanol for several minutes, freeze dried, mounted on aluminum stubs, sputter-coated with gold-palladium, and observed with a JEOL JSM-6060LV scanning electron microscope at 15 kV accelerating voltage.

The terminology follows Neuhaus and Higgins (2002), Sørensen and Pardos (2008), and Neuhaus (2013). Type material has been deposited in the University Museum, University of the Ryukyus (Fujukan), under accession numbers having the prefix RUMF-ZK-00001-00018.

## Results

### Order Cyclorhagida Zelinka, 1896
Family Echinoderidae Bütschli, 1876
Genus *Echinoderes* Claparède, 1863

#### Echinoderes komatsui sp. n.

http://zoobank.org/19C1A89B-5AB4-4886-BE01-B04D55DEEBD1

http://species-id.net/wiki/Echinoderes_komatsui

**Figure 2.**
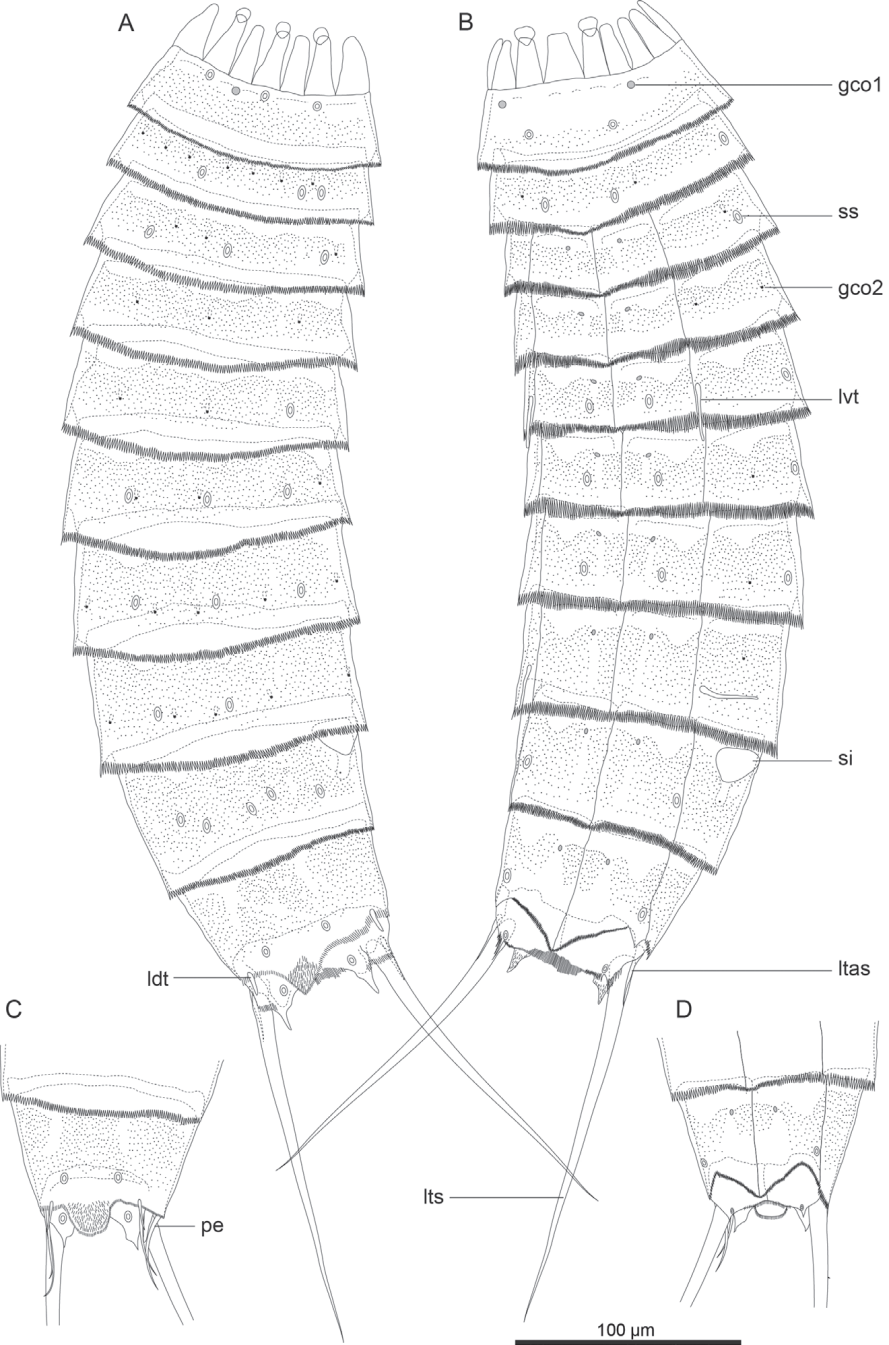
*Echinoderes komatsui* sp. n., camera lucida drawings. **A, B** Holotype, female (RUMF-ZK-00001), entire animal, dorsal and ventral views, respectively **C, D** allotype, male (RUMF-ZK-00002), segments 9–11, dorsal and ventral views, respectively. Double circle, grey circle, and black circle indicate sensory spot, type 1 glandular cell outlet, and type 2 glandular cell outlet, respectively. Abbreviations: gco1, type 1 glandular cell outlet; gco2, type 2 glandular cell outlet; ldt, laterodorsal tubule; ltas, lateral terminal accessory spine; lts, lateral terminal spine; lvt, lateroventral tubule; pe, penile spine; si, sieve plate; ss, sensory spot.

[New Japanese name: Komatsu togekawa] Figs 2 [Fig F3] [Fig F4] [Fig F5] –6

##### Material.

Holotype: Adult female (RUMF-ZK-00001), collected on 26 May 2013 at station 1 (Fig. 1A) by Dr H. Komatsu (National Museum of Nature and Science, Tokyo, Japan) during a sampling cruise with TR/V *Toyoshio-maru* (Hiroshima University, Japan); mounted in Fluoromount G®.

Allotype: Adult male (RUMF-ZK-00002), collected at the same locality as the holotype; mounted in Fluoromount G®.

Paratypes: Three adult females and two adult males RUMF-ZK-00003-00007); two exoskeletons (RUMF-ZK-00008-00009) from DNA-extracted specimens (one adult female and one adult male); all collected at same locality as the holotype; all mounted in Fluoromount G®. Paratype RUMF-ZK-00003 was collected on 26 May 2013, and the others on 8 July 2013.

Additional material: Six specimens for SEM (one adult female, three adult males, and two adults gender undetermined), collected at same locality as holotype on 8 July 2013, mounted on aluminum stubs.

Sequences: 18S sequence (1778 bp) for paratype RUMF-ZK-00008, GenBank accession AB899164; 28S sequence (3292 bp) for paratype RUMF-ZK-00008, GenBank AB899165; COI sequence (658 bp)for paratype RUMF-ZK-00008, GenBank AB899166.

##### Diagnosis.

*Echinoderes* without acicular spines; lateroventral tubules present on segments 5 and 8, laterodorsal tubules on segment 10, and large, sieve plates on segment 9 with an inverted triangular or oval shape; females with lateral terminal accessory spines. Pectinate fringe of the sternal plate on segment 10 with short tips.

##### Description.

**Figure 3. F3:**
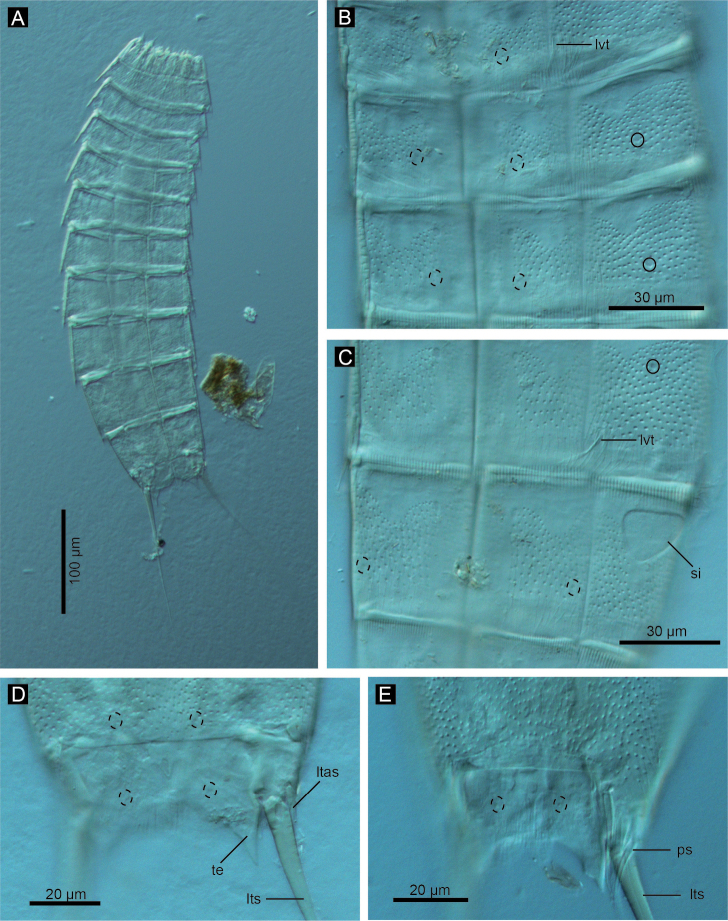
*Echinoderes komatsui* sp. n., Nomarski photomicrographs. **A** Entire animal **B** segments 5–7, ventral view **C** segments 8 and 9, ventral view **D** segments 10 and 11 of female, dorsal view **E** segments 10 and 11 of male, dorsal view. Complete circles indicate type 2 glandular cell outlet; dashed circles indicate sensory spots. Abbreviations: ltas, lateral terminal accessory spine; lts, lateral terminal spine; lvt, lateroventral tubule; ps, penile spine; si, sieve plate; te, tergal extension.

**Figure 4. F4:**
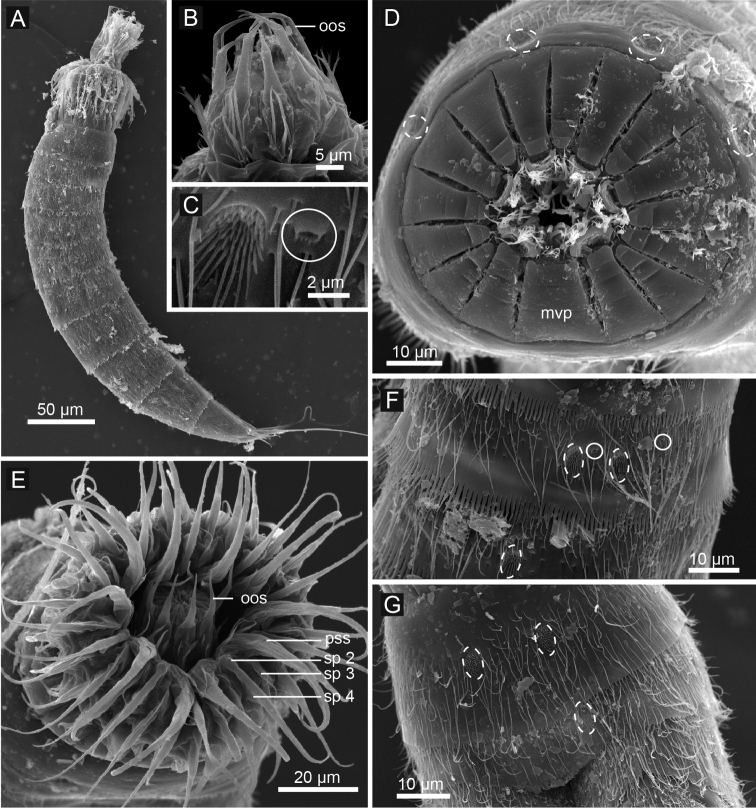
*Echinoderes komatsui* sp. n., scanning electron micrographs. **A** Entire animal, lateral view **B** outer oral styles, lateral view **C** close up of laterodorsal type 2 glandular cell outlet on segment 2 **D** neck, overview **E** partly retracted head, overview **F** segments 2–3, lateral view **G** segments 1–3, ventral view. Complete circles indicate type 2 glandular cell outlets; dashed circles indicate sensory spots. Abbreviations: mvp, midventral placid; oos, outer oral style; pss, primary spinoscalid; sp, spinoscalid followed by ring number.

Adult with head, neck, and eleven trunk segments (Figs 2A, B, 3A, 4A). See Table 2 for measurements. Table 3 indicates the positions of cuticular structures (sensory spots, glandular cell outlets, and tubules).

**Table 2.** Measurements for adult *Echinoderes komatsui* sp. n. (in micrometers). Columns *N* and SD show sample size and standard deviation, respectively. Abbreviations: (f), female condition of sexually dimorphic character; LD, length of laterodorsal tubule; LTAS, length of lateral terminal accessory spine; LTS, length of lateral terminal spine; LV, length of lateroventral tubule; (m), male condition of sexually dimorphic character; MSW, maximum sternal width; S, segment length; SW, standard width; TL, trunk length.

Character	N	Range	Mean	SD
TL	9	304–419	354	45.37
MSW-9	7	49–75	63	11.48
MSW-9/TL	7	15.3–19.8%	17.1%	1.54%
SW-10	7	40–72	58	11.81
SW-10/TL	7	12.7–17.7%	15.6%	1.72%
S1	7	25–41	36	5.82
S2	7	20–35	29	6.27
S3	7	24–33	29	3.97
S4	7	27–37	32	4.42
S5	7	28–38	33	3.83
S6	7	32–42	37	4.15
S7	7	38–48	42	4.47
S8	7	41–55	48	5.55
S9	7	44–59	52	6.39
S10	7	40–50	43	3.54
S11	7	26–38	33	5.48
LV 5	9	19–31	24	4.44
LV 8	9	17–28	23	4.49
LD 10 (m)	4	21–23	22	0.96
LD 10 (f)	5	8–13	10	2.07
LTS	8	145–164	153	6.23
LTS/TL	8	36.6–46.9%	42.3%	3.96%
LTAS (f)	5	12–23	18	5.54

**Table 3.** Summary of location of cuticular structures, tubules, and spines in *Echinoderes komatsui* sp. n. Abbreviations: (f), female condition of sexually dimorphic character; gco1, type 1 glandular cell outlet; gco2, type 2 glandular cell outlet; LD, laterodorsal; ltas, lateral terminal accessory spine; lts, lateral terminal spine; LV, lateroventral; (m), male condition of sexually dimorphic character; MD, middorsal; ML, midlateral; pe, penile spine; SD, subdorsal; si, sieve plate; ss, sensory spot; tu, tubule; VL, ventrolateral; VM, ventromedial.

Position	MD	SD	LD	ML	LV	VL	VM
segment							
1	gco1	ss	ss		gco1	ss	
2	ss	gco2, gco2	ss, gco2, ss, gco2		gco2	ss	
3		gco2, ss		ss, gco2			gco1
4		gco2	gco2		gco2		gco1
5		gco2	ss		tu		gco1, ss
6		gco2, ss	ss	gco2			gco1, ss
7		gco2, ss	gco2, ss	gco2			gco1, ss
8		gco2, ss	gco2	gco2	tu		gco1
9		ss, ss	ss	si		ss	gco1
10		ss	tu			ss	gco1
11		ss		ltas (f), pe (m)	lts	ss	

**Figure 5. F5:**
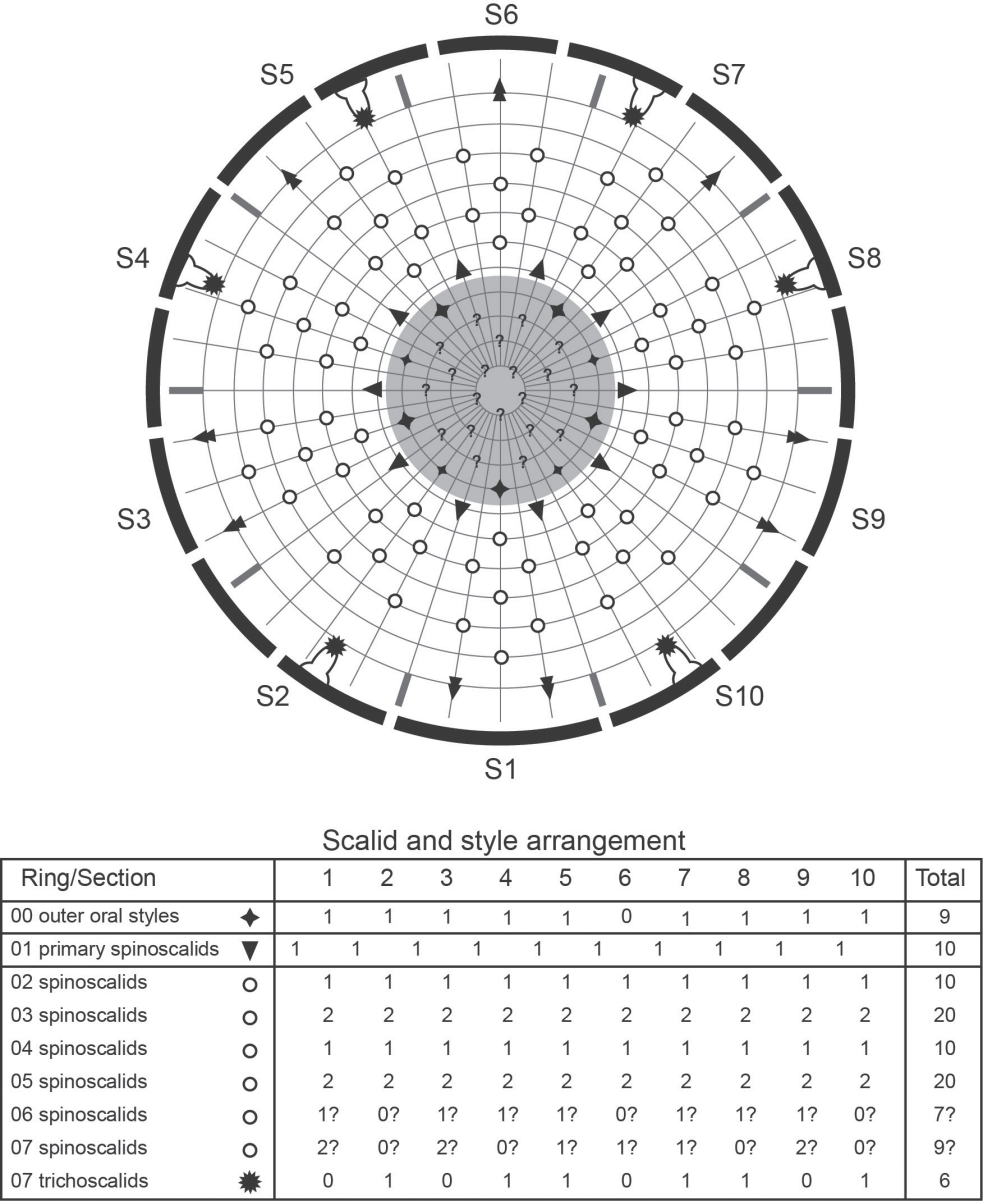
Diagram of mouth cone, introvert, and placids in *Echinoderes komatsui* sp. n. Grey area and heavy line arcs show mouth cone and placids, respectively. The table lists the scalid arrangement by sector.

Head consists of retractable mouth cone and introvert (Figs 4E, 5). Mouth cone with inner oral styles and nine outer oral styles. Exact number and arrangement of inner oral styles not examined. Each outer oral style consists of rectangular basal part and triangular distal part (Fig. 4B). Basal parts of outer oral styles alternating in size: five large in odd sectors of introvert, and four small in even sectors (Figs 4B, 5). Introvert composed of seven rings of spinoscalids and one ring of trichoscalids (Figs 4E, 5). Ring 01 includes ten primary spinoscalids each with basal sheath and smooth long end piece (Fig. 4E). Each basal sheath with three overlapping fringes. Proximal fringe extends into three flat projections, like a trident, covering next fringe. Middle fringe with two lateral projections overlapping end piece. Distal fringe with five threads projecting between two projections of middle fringe. End piece of primary spinoscalids is longest unit. Rings 02 and 04 with 10 spinoscalids; rings 03 and 05 with 20 spinoscalids. Spinoscalids of rings 02–05 equal length. Rings 06 and 07 not examined in detail, but ring 06 with at least seven relatively short spinoscalids, and ring 07 with nine leaf-like scalids (Fig. 5). Six trichoscalids attached with trichoscalid plate in sectors 2, 4, 5, 7, 8, and 10.

Neck with 16 placids (Figs 2A, B, 4D, 5). Midventral placid broadest (ca. 18 μm at basal width and ca. 10 μm at tip width). Remaining placids similar in size, but differ alternately in tip width (ca. 12 μm at basal width and ca. 5–6 μm at tip width) (Fig. 4D).

Segment 1 consists of complete cuticular ring with thick pachycyclus at anterior margin. Bracteate cuticular hairs densely cover entire dorsal side, and posterior area of ventral side (Fig. 2A, B). Rounded subdorsal and laterodorsal sensory spots located close to anterior margin of the segment (Fig. 2A). Rounded ventrolateral sensory spots located central between anterior and posterior segment margins (Figs 2B, 4G). Type 1 glandular cell outlets situated anteriorly in middorsal and lateroventral positions (Fig. 2A, B). Posterior part of the segment with pectinate fringe showing longer fringe tips laterally (Fig. 2A, B).

Segment 2 with complete cuticular ring, like segment 1 (Fig. 2A, B). This and following eight segments with thick pachicycli at anterior margins. Bracteate cuticular hairs densely cover whole area. One oval sensory spot in middorsal position, two pairs in laterodorsal position, and one pair in ventrolateral position (Figs 2A, B, 4F, G). All sensory spots central in position. Two pairs of type 2 glandular cell outlets in both subdorsal and laterodorsal positions (Figs 2A, B, 4C, F). Pair of type 2 glandular cell outlets in lateroventral position. All type 2 glandular cell outlets of segment 2 and following six segments situated centrally of the segment. In LM observation, type 2 glandular cell outlets show funnel shaped structure, whereas in SEM observation, they show single small pore in slightly protruded cuticular surface (Fig. 4C). Posterior margin of the segment ends as pectinate fringe showing longer tips than tips of preceding segment (Figs 2A, B, 4F).

Segment 3 and following eight segments consist of one tergal and two sternal plates (Fig. 2A, B). This and following seven segments entirely covered with bracteate cuticular hairs except for anterior area (Figs 2A, B, 3B, C). Paired sensory spots in subdorsal and midlateral positions (Figs 2A, B, 4F). Type 1 glandular cell outlets of segment 3 and following seven segments situated at anterior part of segment in ventromedial position (Fig. 2B). Pair of type 2 glandular cell outlets in subdorsal and midlateral positions (Fig. 2A, B). Pectinate fringe on segment 3 and five following segments as on segment 2.

Segment 4 without sensory spots. Type 2 glandular cell outlets in subdorsal, laterodorsal, and lateroventral positions (Fig. 2A, B).

**Figure 6.**
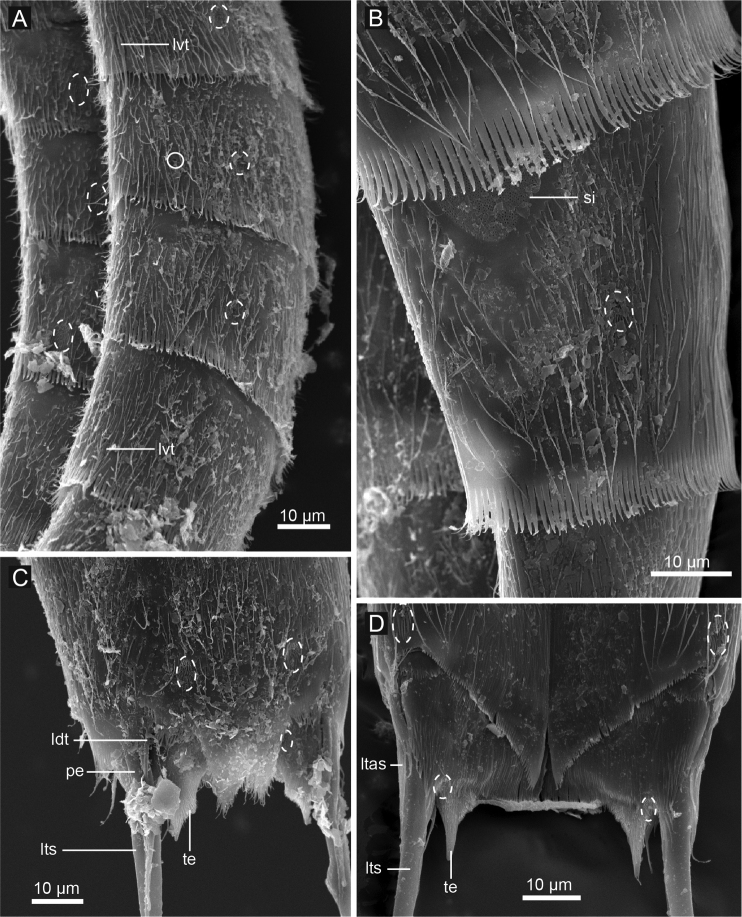
*Echinoderes komatsui* sp. n., scanning electron micrographs. **A** Segments 5–8, lateral view **B** segments 8, lateral view **C** segments 10 and 11, male, dorsal view **D** segments 10 and 11, female, ventral view. Complete circle indicates type 2 glandular cell outlet; dashed circles indicate sensory spots. Abbreviations: ldt, laterodorsal tubule; ltas, lateral terminal accessory spine; lts, lateral terminal spine; lvt, lateroventral tubule; pe, penile spine; si, sieve plate; te, tergal extension.

Segment 5 with lateroventral tubules (Figs 2B, 3B, 6A). Sensory spots in laterodorsal and ventromedial positions (Figs 2A, B, 3B, 6A). Paired type 2 glandular cell outlets in subdorsal position (Fig. 2A).

Segment 6 with subdorsal, laterodorsal, and ventromedial paired sensory spots (Figs 2A, B, 3B, 6A). Paired type 2 glandular cell outlets in subdorsal and midlateral positions (Figs 2A, B, 3B, 6A).

Segment 7 similar to segment 6 but with additional laterodorsal type 2 glandular cell outlets (Figs 2A, B, 3B, 6A).

Segment 8 with lateroventral tubules (Figs 2B, 3C, 6A). Paired sensory spots in subdorsal position (Fig. 2A). Positions of type 2 glandular cell outlets as on segment 7.

Segment 9 with two pairs of subdorsal sensory spots and one pair of laterodorsal and ventrolateral sensory spots (Figs 2A, B, 3C, 6B). Pair of sieve plates with wide sieve area and single posterior pore situated in midlateral position. Sieve area variable in shape, forming inverted triangle in some specimens and broad oval in others (Figs 2A, B, 3C, 6B). Tips of pectinate fringe slightly shorter in length than on preceding segment.

Segment 10 with thin laterodorsal tubules (Figs 2A, 6C). Length of laterodorsal tubules in males about twice as long as those in females. Paired subdorsal and ventrolateral sensory spots situated close to posterior margin of segment (Figs 2, 3D, 6C, D). Posterior middorsal margin elongated, extending to segment 11 in some specimens (Figs 2A, 6C), but truncate in other specimens. Posterior margin ends as pectinate fringe with short tips, except in ventrolateral area, which without pectination.

Segment 11 with lateral terminal spines (Figs 2, 3D, E, 6C, D). Short, thin lateral terminal accessory spines present only in females (Figs 2B, 3D, 6D), and three pairs of penile spines only in males (Figs 2C, 3E, 6C). Cuticular hairs absent. Paired sensory spots present in subdorsal position at base of tergal extension (Figs 2A, C, 3D, E, 6C). Ventrolateral paired sensory spots placed close to posterior margin of sternal plate (Figs 2B, D, 6D). Tergal plate projects laterally and ends in short, pointed tergal extensions (Figs 2A, C, 3D, E, 6C, D).

##### Etymology.

The species is named after Dr H. Komatsu (National Museum of Nature and Science, Tokyo, Japan), a taxonomist of brachyuran crabs and the first person to find *Echinoderes komatsui* sp. n.

##### Remarks.

Among congeners, *Echinoderes komatsui* sp. n. is most similar to *Echinoderes applicitus*, *Echinoderes coulli*, and *Echinoderes marthae* in sharing the following combination of characters: (1) lateral (lateroventral or lateral accessory) tubules only on segments 5 and 8, (2) middorsal spines absent on all segments, and (3) large sieve plates present (Higgins 1977, Ostmann et al. 2012, Sørensen 2014). *Echinoderes komatsui* sp. n. additionally shares with *Echinoderes applicitus* a pair of laterodorsal tubules and a posteriorly extended margin of the tergal plate on segment 10. The latter character, previously considered diagnostic for *Echinoderes applicitus*, is variable in *Echinoderes komatsui* sp. n. *Echinoderes komatsui* sp. n. differs from *Echinoderes applicitus* in having (1) lateral terminal accessory spines in females, (2) type 2 glandular cell outlets on several segments, and (3) a pectinate fringe with short, narrow tips on the sternal plate of segment 10 (long wide tips in *Echinoderes applicitus*).

*Echinoderes komatsui* sp. n. is identical to *Echinoderes coulli* in the formula of ventral tubules, and both trunk lengths overlap (304–419 μm in *Echinoderes komatsui* sp. n.; 248–364 μm in *Echinoderes coulli*). *Echinoderes komatsui* sp. n. differs from *Echinoderes coulli* in having (1) laterodorsal tubules on segment 10, (2) lateral terminal accessory spines in females, and (3) much longer lateral terminal spines (145–164 μm and 36.6–46.9% of trunk length in *Echinoderes komatsui* sp. n.; 18–68 μm and 5.5–23.9% of trunk length in *Echinoderes coulli*).

*Echinoderes komatsui* sp. n. shares with *Echinoderes marthae* the presence of tubules on segment 8, however, the number of tubules is different. *Echinoderes komatsui* sp. n. has only lateroventral tubules, whereas *Echinoderes marthae* has lateroventral and laterodorsal tubules. Furthermore, *Echinoderes komatsui* sp. n. differs from *Echinoderes marthae* in having (1) type 2 glandular cell outlets on some segments, (2) lateral terminal accessory spines in females, and (3) three pairs of penile spines in males (two pairs in *Echinoderes marthae*).

#### Echinoderes hwiizaa sp. n.

http://zoobank.org/64C79CE3-861E-43BE-B7BC-F3F0FE6819EA

http://species-id.net/wiki/Echinoderes_hwiizaa

**Figure 7.**
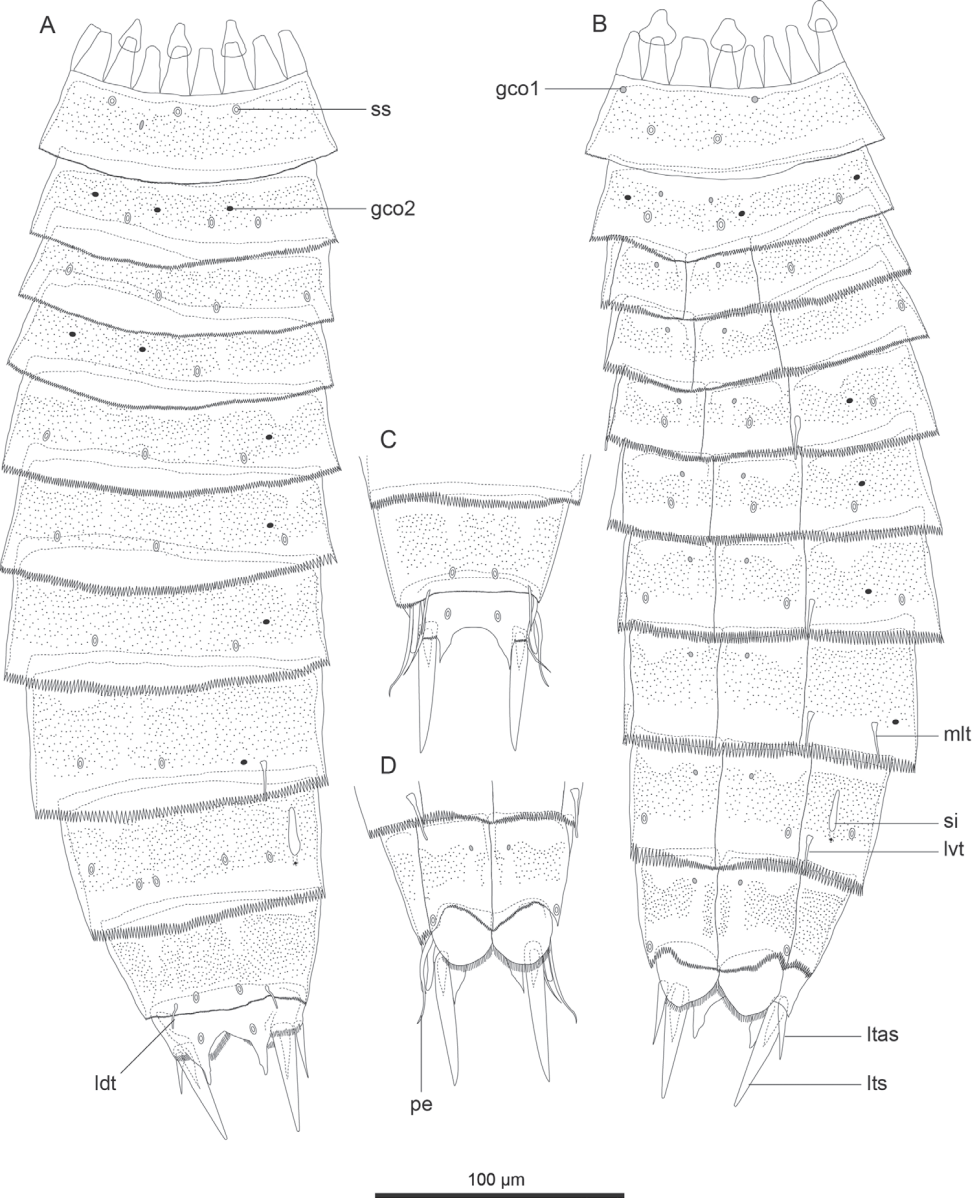
*Echinoderes hwiizaa* sp. n., camera lucida drawings. **A, B** paratype, female (RUMF-ZK-00016), entire animal, dorsal and ventral views, respectively **C, D** allotype, male (RUMF-ZK-00011), segments 9–11, dorsal and ventral views, respectively. Double circle, grey circle, and black circle indicate sensory spot, type 1 glandular cell outlet, and type 2 glandular cell outlet, respectively. Abbreviations: gco1, type 1 glandular cell outlet; gco2, type 2 glandular cell outlet; ldt, laterodorsal tubule; ltas, lateral terminal accessory spine; lts, lateral terminal spine; lvt, lateroventral tubule; mlt, midlateral tubule; pe, penile spine; si, sieve plate; ss, sensory spot.

[New Japanese name: Yagitsuno togekawa] Figs 7 [Fig F8] [Fig F9] [Fig F10] –11

##### Material.

Holotype (RUMF-ZK-00010): Adult female, collected by H. Yamasaki on 23 June 2013 at station 2 (Fig. 1B); mounted in Fluoromount G®.

Allotype (RUMF-ZK-00011): Adult male, collected at the same locality as the holotype; mounted in Fluoromount G®.

Paratypes: Two adult females and two adult males (RUMF-ZK-00012-00015); three exoskeletons (RUMF-ZK-00016-00018) from specimens used for DNA extraction (one adult female and two adult males); all collected at the same locality as the holotype; all mounted in Fluoromount G®.

Other material: six specimens for SEM (four adult females, one adult male, and one adult gender undetermined), collected at the same locality as the holotype, mounted on aluminum stubs.

Sequences: 18S sequence (1775 bp) for paratype RUMF-ZK-00017, Genbank accession AB899167; 28S sequence (2233 bp) for paratype RUMF-ZK-00017, GenBank AB899168; COI sequences (all 658 bp) for three paratypes (RUMF-ZK-00016-00018), GenBank AB899169–AB899171, respectively.

##### Diagnosis.

*Echinoderes* without acicular spines; with lateroventral tubules on segments 5, 7, 8, and 9, midlateral tubules on segment 8, laterodorsal tubules on segment 10, and large, narrow oval-shaped sieve plates on segment 9; lateral terminal spines relatively thick, short, with blunt tips, length about 10–15% of trunk length.

##### Description.

**Figure 8. F8:**
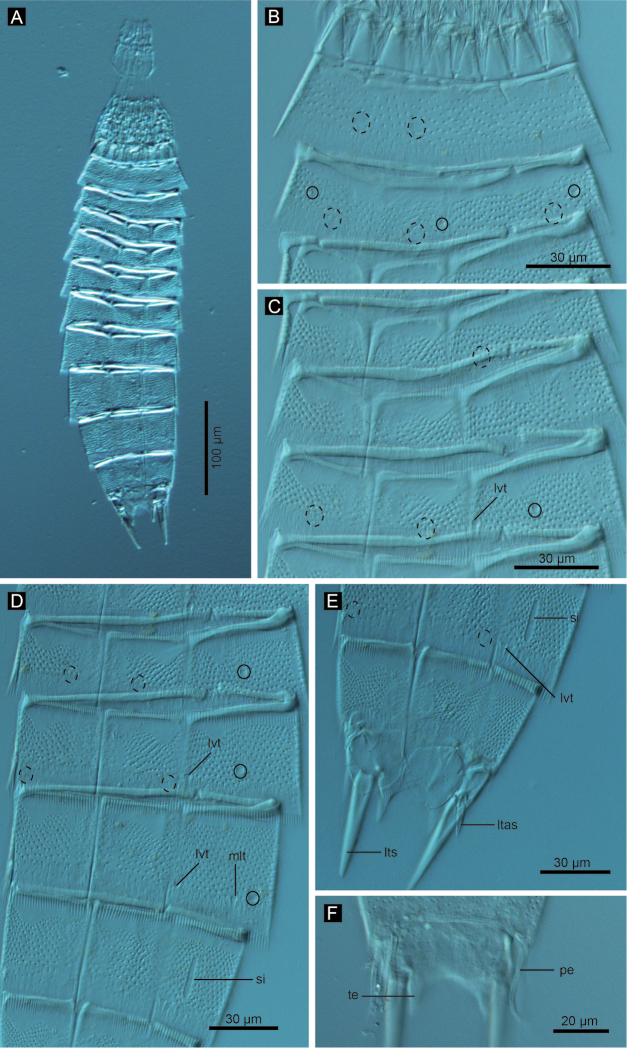
*Echinoderes hwiizaa* sp. n., Nomarski photomicrographs. **A** entire animal **B** segments 1–2, ventral view **C** segments 3–5, ventral view **D** segments 6–9 ventral view **E** segments 9–11 of female, ventral view **F** segment 11 of male, dorsal view. Complete circles indicate type 2 glandular cell outlet; cashed circles indicate sensory spots. Abbreviations: ltas, lateral terminal accessory spine; lts, lateral terminal spine; lvt, lateroventral tubule; mlt, midlateral tubule; pe, penile spine; si, sieve plate; te, tergal extension.

Adult with head, neck and eleven trunk segments (Figs 7A, B, 8A). See Table 4 for measurements, and Table 5 for positions of cuticular structures (sensory spots, glandular cell outlets, and tubules).

**Table 4.** Measurements for adult *Echinoderes hwiizaa* sp. n. (in micrometers). Columns N and SD show sample size and standard deviation, respectively. Abbreviations: (f), female condition of sexually dimorphic character; LD, length of laterodorsal tubule; LTAS, length of lateral terminal accessory spine; LTS, length of lateral terminal spine; LV, length of lateroventral tubule; (m), male condition of sexually dimorphic character; ML, length of midlateral tubule; MSW, maximum sternal width; S, segment length; SW, standard width; TL, trunk length.

Character	N	Range	Mean	SD
TL	8	385–414	400	11.72
MSW-8	9	64–75	69	3.99
MSW-8/TL	8	16.7–19.1%	17.4%	0.75%
SW-10	9	52–65	61	4.08
SW-10/TL	8	13.5–16.1%	15.2%	0.89%
S1	8	38–47	41	2.53
S2	8	30–41	36	3.66
S3	9	28–32	30	1.56
S4	9	31–33	32	0.71
S5	9	31–38	34	1.79
S6	9	37–43	39	1.66
S7	9	43–48	46	1.94
S8	9	46–56	52	3.45
S9	9	46–56	50	3.2
S10	9	43–52	46	3.42
S11	9	34–46	38	4.16
LV 5	9	18–25	20	2.1
LV 7	9	15–22	34	2.42
ML 8	9	16–22	19	2.36
LV 8	9	16–22	19	1.81
LV 9	9	13–19	17	1.71
LD 10 (m)	5	15–21	18	2.39
LD 10 (f)	4	10–14	12	2.06
LTS	9	46–53	51	2.11
LTS/TL	9	11.6–13.5%	12.7%	0.59%
LTAS (f)	4	21–29	26	3.58

**Table 5.** Summary of location of cuticular structures, tubules, and spines in *Echinoderes hwiizaa* sp. n. Abbreviations: (f), female condition of sexually dimorphic character; gco1, type 1 glandular cell outlet; gco2, type 2 glandular cell outlet; LD, laterodorsal; ltas, lateral terminal accessory spine; lts, lateral terminal spine; LV, lateroventral; (m), male condition of sexually dimorphic character; MD, middorsal; ML, midlateral; PD, paradorsal; pe, penile spine; SD, subdorsal; si, sieve plate; SL, sublateral; ss, sensory spot; tu, tubule; VL, ventrolateral; VM, ventromedial.

Position	MD	PD	SD	LD	ML	SL	LV	VL	VM
segment									
1	gco1		ss	ss			gco1		ss
2	ss		gco2	ss, gco2, ss				gco2	ss, gco1
3			ss	ss		ss			gco1
4			gco2	ss					gco1
5			ss	ss	gco2		tu		gco1, ss
6			ss		gco2, ss				gco1, ss
7	ss			ss	gco2		tu	ss	gco1
8			ss	gco2	tu		tu		gco1
9		ss		ss	ss	si	tu	ss	gco1
10			ss	tu				ss	gco1
11			ss		ltas (f), pe (m)		lts		

**Figure 9. F9:**
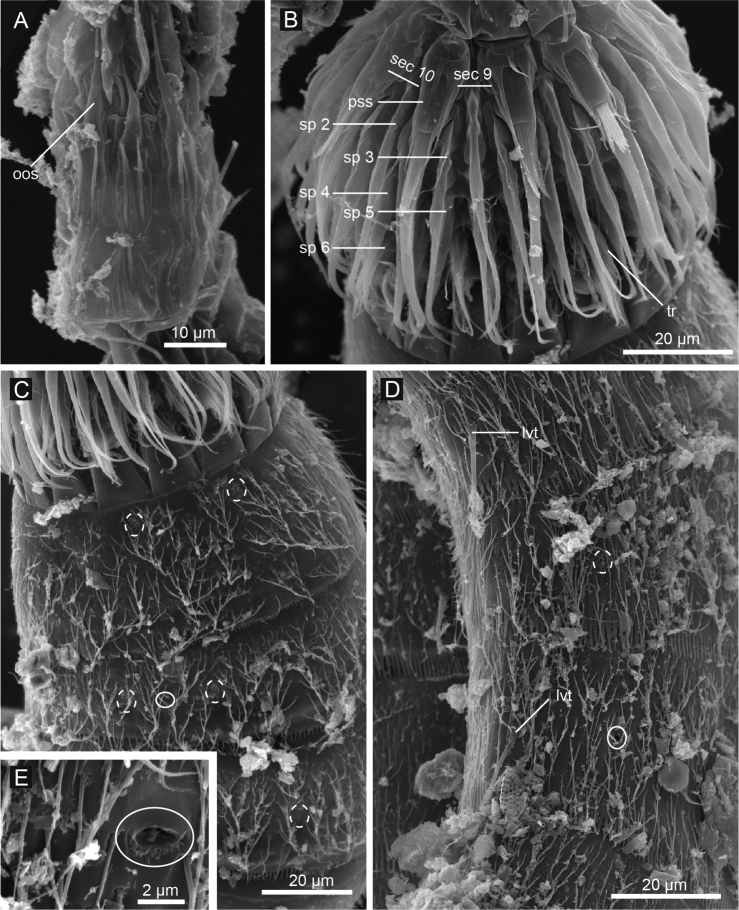
*Echinoderes hwiizaa* sp. n., scanning electron micrographs. **A** Mouth cone **B** introvert, lateral view **C** neck and segments1–3, lateral view **D** segments 5–7, lateral view **E** close up of laterodorsal type 2 glandular cell outlet on segment 2. Complete circles indicate type 2 glandular cell outlet; dashed circles indicate sensory spots. Abbreviations: lvt, lateroventral tubule; oos, outer oral style; pss, primary spinoscalid; sec, introvert sector followed by sector number; sp, spinoscalid followed by ring number.

**Figure 10. F10:**
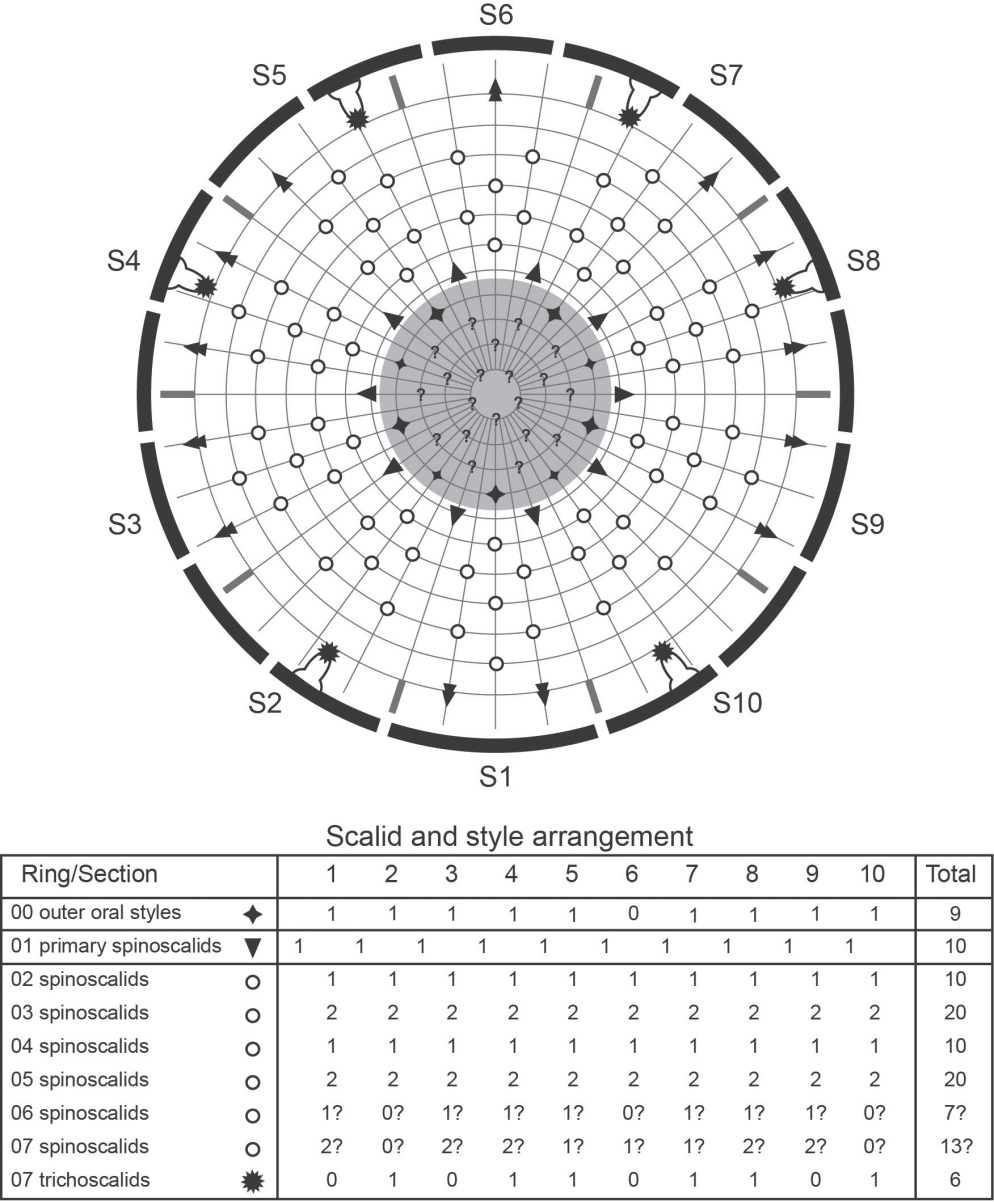
Diagram of mouth cone, introvert, and placids in *Echinoderes hwiizaa* sp. n. Grey area and heavy line arcs show mouth cone and placids, respectively. The table lists the scalid arrangement by sector.

Head consists of retractable mouth cone and introvert (Figs 9A, B, 10). Mouth cone with inner oral styles and nine outer oral styles. Exact number and arrangement of inner oral styles not observed. Each outer oral style composed of rectangular basal part and triangular distal part. Basal parts of outer oral styles alternate in size: five large in odd sectors of introvert, and four small in even sectors (Fig. 9A). Posterior to basal part of each outer oral style, two spinose hairs project anteriorly, covering outer oral style (Fig. 9A). Introvert composed of seven rings of scalids and one ring of trichoscalids (Figs 9B, 10). Ring 01 includes ten primary spinoscalids with basal sheath and long, smooth end piece (Fig. 9B). Each basal sheath with three fringes. Proximal fringe extends into three long projections, like a trident, covering next fringe. Middle basal fringe with two lateral projections, overlapping end piece. Distal fringe with five to seven threads projecting between two projections of middle fringe. End piece of primary spinoscalids is longest unit. Rings 02 and 04 with 10 spinoscalids, and rings 03 and 05 with 20 spinoscalids. Spinoscalids of rings 02–05 similar in length. Rings 06 and 07 could not be examined in detail, but at least seven relatively short spinoscalids present in ring 06, and 13 leaf-like scalids in ring 07. Six trichoscalids present each attached with trichoscalid plate in sectors 2, 4, 5, 7, 8, and 10.

Neck with 16 placids (Figs 7A, B, 8B, 10). Midventral placid broadest (ca. 17 μm at basal width and ca. 11 μm at tip width); remaining placids with similar size (ca. 11 μm at basal width and ca. 5 μm at tip width).

Segment 1 consists of complete cuticular ring with pachycyclus at anterior margin (Figs 7A, B, 8B). Non-bracteate cuticular hairs densely cover entire segment (Fig. 7A, B). Paired rounded subdorsal and laterodorsal sensory spots located close to anterior margin of the segment (Figs 7A, 9C). Rounded ventromedial sensory spots centered between anterior and posterior margins (Fig. 7B). Type 1 glandular cell outlets situated anteriorly in middorsal and lateroventral positions (Fig. 7A, B). Posterior part of the segment with pectinate fringe with very short tips (Fig. 7A, B).

Segment 2 also with complete cuticular ring (Fig. 7A, B), with thick pachycyclus at anterior margin (Fig. 8B). All cuticular surface, except anterior and posterior areas covered with bracteate cuticular hairs (Figs 7A, B, 8B, 9C). Oval sensory spots in middorsal, two pairs in laterodorsal, and pair in ventromedial positions (Figs 7A, B, 8B, 9C). Type 2 glandular cell outlets in subdorsal, laterodorsal, and ventrolateral positions (Figs 7A, B, 9C). All type 2 glandular cell outlets of this segment and segment 4–7 situated slightly anterior to sensory spots. In LM observation, type 2 glandular cell outlets show oval or box shaped structure, whereas in SEM observation, they show single large pore (Fig. 9E). Type 1 glandular cell outlets placed close to anterior margin in ventromedial position on this and following eight segments (Fig. 7A, B). Posterior margin of segment with pectinate fringe with longer tips than on preceding segment (Figs 7A, B, 9C).

Segment 3 and following eight segments consist of one tergal and two sternal plates (Fig. 7A, B). Each plate with thicker pachycycli in anterior areas and articulate areas with other plates. Cuticular hairs on this and following seven segments bracteate, covering entire segment except in anterior, posterior, and paraventral areas (Fig. 7A, B). Sensory spots in subdorsal, laterodorsal, and sublateral positions (Figs 7A, B, 8C). Pectinate fringes as on segment 2.

Segment 4 with pair of laterodorsal sensory spots and paired subdorsal type 2 glandular cell outlets (Fig. 7A, B). Pectinate fringes as on segment 2.

Segment 5 with lateroventral tubules (Figs 7B, 8C, 9D). Paired sensory spots in subdorsal, laterodorsal, and ventromedial positions (Figs 7A, B, 8C). Pair of type 2 glandular cell outlets located in midlateral position (Figs 7A, B, 8C). Tips of pectinate fringes similar in length, and longer than those on three preceding segments on this and following four segments.

Segment 6 with paired subdorsal, midlateral, and ventromedial sensory spots (Figs 7A, 8D, 9D). Pair of type 2 glandular cell outlets present in midlateral position (Figs 7A, B, 8D).

Segment 7 with lateroventral tubules (Figs 7B, 8D, 9D). Middorsal and paired laterodorsal and ventrolateral sensory spots present (Figs 7A, B, 8D). Type 2 glandular cell outlets in midlateral position (Figs 7A, B, 8D, 9D).

**Figure 11.**
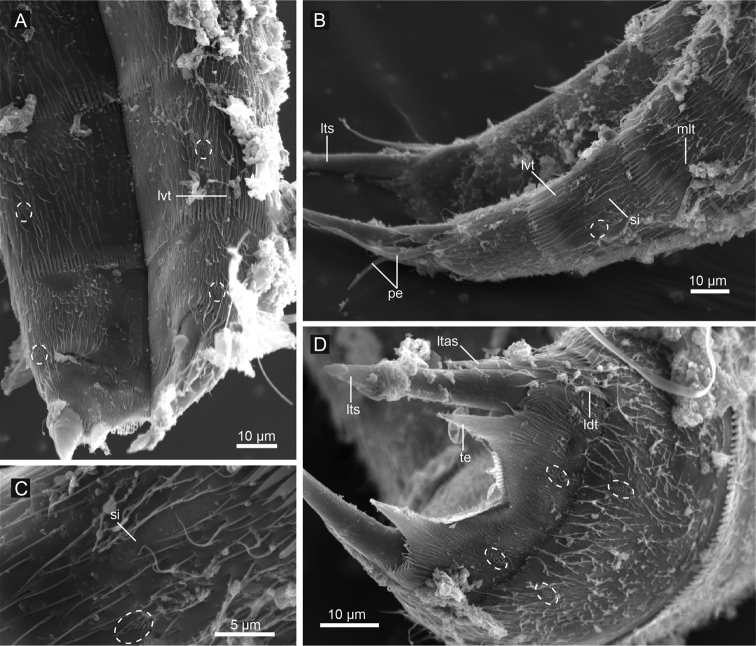
*Echinoderes hwiizaa* sp. n., scanning electron micrographs. **A** segments 8–11, ventral view **B** segments 8–11, lateral view **C** close up showing sieve plate on segment 9 **D** segments 10 and 11, female, dorsal view. Dashed circles indicate sensory spots. Abbreviations: ldt, laterodorsal tubule; ltas, lateral terminal accessory spine; lts, lateral terminal spine; lvt, lateroventral tubule; mlt, midlateral tubule; pe, penile spine; si, sieve plate; te, tergal extension.

Segment 8 with midlateral and lateroventral tubules (Figs 7A, B, 8D, 11B). Paired sensory spots in subdorsal position (Fig. 7A). Paired type 2 glandular cell outlets in laterodorsal position, close to midlateral tubules (Figs 7A, B, 8D).

Segment 9 with lateroventral tubules (Figs 7B, 8E, 11A, B). Paired paradorsal, laterodorsal, midlateral, and ventrolateral sensory spots present (Figs 7A, B, 8E, 11A, B, C). Sieve plates with narrow, oval sieve area and single posterior pore present in sublateral position (Figs 7A, B, 8D, E, 11B, C).

Segment 10 with thin laterodorsal tubules in males, and short, thin, hook-shaped laterodorsal tubules in females (Figs 7A, C, 11D). Paired subdorsal and ventrolateral sensory spots situated close to posterior margin of the segment (Figs 7A–D, 11D). Posterior margin ends as pectinate fringe with short tips.

Segment 11 with short and thick lateral terminal spines ending in blunt tip (Figs 7A–D, 8E, 11D). Pair of short lateral terminal accessory spines present only in females (Figs 7A, B, 8E, 11D), and three pairs of penile spines present only in males (Figs 7C, D, 8F, 11B). Cuticular hairs absent. Paired sensory spots situated in subdorsal position (Figs 7A, C, 11D). Tergal plate projects laterally and ends in short, pointed tergal extensions (Figs 7A–D, 8F, 11D).

##### Etymology.

The species name comes from *hwiizaa* (‘goat’) from one of the Okinawan local languages, referring to the short, thick lateral terminal spines that resemble the horns of goat.

**Remarks.** Among *Echinoderes* species, only *Echinoderes hwiizaa* sp. n. and *Echinoderes marthae* (Sørensen 2014) have two pairs of tubules on segment 8 and lack dorsal acicular spines. *Echinoderes hwiizaa* sp. n. differs from *Echinoderes marthae* in having (1) lateroventral tubules on segments 7 and 9, (2) very short, thick, blunt lateral terminal spines (46–53 μm long and 11.6–13.5% of trunk length in *Echinoderes hwiizaa* sp. n.; 74–103 μm long and 20.4–33.2% of trunk length in *Echinoderes marthae*), (3) lateral terminal accessory spines in females, and (4) three pairs of penile spines in males (two pairs in male *Echinoderes marthae*).

## Discussion

The *Echinoderes coulli* group previously accommodated seven species: *Echinoderes coulli*, *Echinoderes applicitus*, *Echinoderes marthae*, *Echinoderes maxwelli*, *Echinoderes ohtsukai*, *Echinoderes rex*, and *Echinoderes teretis* (Omer-Cooper 1957, Higgins 1977, Brown 1985, Lundbye et al. 2011, Ostmann et al. 2012, Yamasaki and Kajihara 2012). These species share the following characters: (1) absence of middorsal acicular spines, or presence of a single spine on segment 4; (2) absence of lateroventral acicular spines, or presence of very short ones only on segments 6 and 7; (3) lateral tubules on at least segments 5 and 8; (4) relatively large sieve plates consisting of a sieve area and a posterior pore; (5) lateral terminal accessory spines lacking in both sexes. In addition, all species except for *Echinoderes rex* were reported from intertidal brackish habitats, such as intertidal flats or mangrove areas, where other echinoderid species are rarely found. These morphological and ecological similarities have been viewed as an evidence to consider the *Echinoderes coulli* group a monophyletic group.

Both their morphological characters and habitats suggest that *Echinoderes komatsui* sp. n. and *Echinoderes hwiizaa* sp. n. are closely related to the seven species which are known as members of the *Echinoderes coulli* group, and the two new species also belong to the group. Two new species both lack spines on segments 1–10 completely, have lateroventral tubules on segments 5 and 8 (additionally on segments 7 and 9, and midlateral tubules on segment 8 in *Echinoderes hwiizaa* sp. n.), and have relatively large sieve plates. In addition, *Echinoderes komatsui* sp. n. was collected in a mangrove area and *Echinoderes hwiizaa* sp. n. on an intertidal flat, both areas of variable and often reduced salinity.

*Echinoderes komatsui* sp. n. and *Echinoderes hwiizaa* sp. n. differ from other species in the *Echinoderes coulli* group in one particular feature, namely the lack of lateral terminal accessory spines in females. The former two species possess lateral terminal accessory spines in females, however, these spines are very short and seem to be poorly developed. One possibility is that the previous seven species included in the *Echinoderes coulli* group are more closely related to one another rather than to any of the two new species. However, the relationships within the group have never been examined with a cladistic analysis. Since the relationships within the group are still open to question, the future phylogenetic studies using more abundant morphological data and/or molecular markers are needed.

Below follows a revised diagnosis of the *Echinoderes coulli* group and a dichotomous key to species in the group, modified from Sørensen (2014).

### Diagnosis of *Echinoderes coulli* group

*Echinoderes* without acicular middorsal spines, or with a very short spine only on segment 4; lateral spines very short on segments 6 and 7, or absent; midlateral, sublateral, lateral accessory, or lateroventral tubules on segments 5 and 8; sieve plates relatively large, consisting of oval or inverted-triangular sieve area and single posterior pore; lateral terminal accessory spines poorly developed or completely absent in females.

### Dichotomous key to species in the *Echinoderes coulli* group

**Table:** 

1	Middorsal spine present on segment 4	2
–	Middorsal spines absent	5
2	Lateroventral acicular spines present on segments 6 and 7	3
–	Lateroventral acicular spines absent	4
3	Lateral terminal spines conspicuously short, less than 30 μm long; trunk length more than 400 μm long	*Echinoderes rex*
–	Lateral terminal spines more than 100 μm long; trunk length less than 300 μm long; trunk shows hunch-back-shape	*Echinoderes teretis*
4	Type 2 glandular cell outlets absent; segment 10 projecting over segment 11	*Echinoderes maxwelli*
–	Type 2 glandular cell outlets present; segment 10 not projecting, not reaching posterior margin of segment 11	*Echinoderes ohtsukai*
5	One pair of tubules on segment 8	6
–	Two pairs of tubules on segment 8	8
6	Laterodorsal tubules present on segment 10; lateral terminal spine measuring more than ca. 30% of trunk length	7
–	Laterodorsal tubules absent; lateral terminal spine measuring less than ca. 25% of trunk length	*Echinoderes coulli*
7	Type 2 glandular cell outlets present on several segments; pectinate fringes of ventral side of segment 10 with short, narrow tips; lateral terminal accessory spines present in females	*Echinoderes komatsui* sp. n.
–	Type 2 glandular cell outlets absent; pectinate fringes of ventral side of segment 10 with long, wide tips; lateral terminal accessory spines absent in females	*Echinoderes applicitus*
8	Lateroventral tubules on segments 7 and 9; lateral terminal spines thick, blunt, and measuring less than 15% of trunk length	*Echinoderes hwiizaa* sp. n.
–	Lateroventral tubules absent on segments 7 and 9; lateral terminal spines thin, pointed, and measuring more than 20% of trunk length	*Echinoderes marthae*

## Supplementary Material

XML Treatment for Echinoderes komatsui

XML Treatment for Echinoderes hwiizaa
